# Strain Engineering for Enhancing Carrier Mobility in MoTe_2_ Field‐Effect Transistors

**DOI:** 10.1002/advs.202303437

**Published:** 2023-08-08

**Authors:** Abde Mayeen Shafi, Md Gius Uddin, Xiaoqi Cui, Fida Ali, Faisal Ahmed, Mohamed Radwan, Susobhan Das, Naveed Mehmood, Zhipei Sun, Harri Lipsanen

**Affiliations:** ^1^ Department of Electronics and Nanoengineering Aalto University Tietotie 3 FI‐02150 Finland; ^2^ QTF Centre of Excellence Department of Applied Physics Aalto University Aalto FI‐00076 Finland

**Keywords:** MoTe_2_, Al_2_O_3_, tensile strain, carrier mobility, metal–insulator transition

## Abstract

Molybdenum ditelluride (MoTe_2_) exhibits immense potential in post‐silicon electronics due to its bandgap comparable to silicon. Unlike other 2D materials, MoTe_2_ allows easy phase modulation and efficient carrier type control in electrical transport. However, its unstable nature and low‐carrier mobility limit practical implementation in devices. Here, a deterministic method is proposed to improve the performance of MoTe_2_ devices by inducing local tensile strain through substrate engineering and encapsulation processes. The approach involves creating hole arrays in the substrate and using atomic layer deposition grown Al_2_O_3_ as an additional back‐gate dielectric layer on SiO_2_. The MoTe_2_ channel is passivated with a thick layer of Al_2_O_3_ post‐fabrication. This structure significantly improves hole and electron mobilities in MoTe_2_ field‐effect transistors (FETs), approaching theoretical limits. Hole mobility up to 130 cm^−2^ V^−1^ s^−1^ and electron mobility up to 160 cm^−2^ V^−1^ s^−1^ are achieved. Introducing local tensile strain through the hole array enhances electron mobility by up to 6 times compared to the unstrained devices. Remarkably, the devices exhibit metal–insulator transition in MoTe_2_ FETs, with a well‐defined critical point. This study presents a novel technique to enhance carrier mobility in MoTe_2_ FETs, offering promising prospects for improving 2D material performance in electronic applications.

## Graphical abstract

A deterministic method is presented to enhance MoTe_2_ device performance by inducing tensile strain through substrate engineering and encapsulation. This structure significantly improves hole and electron mobilities in MoTe_2_ field‐effect transistors, reaching up to 130 and 160 cm^2^ V s, respectively. Remarkably, the electron mobility increases by 6 times compared to the unstrained devices. The technique holds potential for 2D‐electronics.

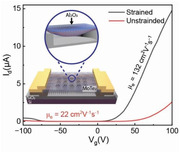

## 1 Introduction

MoTe_2_ has emerged as a promising 2D transition metal dichalcogenide (TMDC) in recent years, owing to its unique properties and potential to replace silicon in various applications.^[^ ^1^, ^2^, ^3^ ^]^ A few‐layer 2H‐MoTe_2_ has an indirect bandgap of around 0.9 eV which makes it promising for applications in state‐of‐the‐art electronic and optoelectronic devices operating in the visible to near‐infrared range.^[^ ^4^, ^5^ ^]^ The weak Fermi‐level pinning in MoTe_2_ and metal contacts allows for easy modulation of device polarity through contact engineering.^[^ ^6^ ^]^ Despite its potential, low carrier mobility and high contact resistance in MoTe_2_ field effect transistors (FETs), along with the material instability in atmospheric conditions, remain major obstacles to its integration into electronic devices.^[^ ^7^, ^8^ ^]^ Theoretically, the room‐temperature hole and electron mobilities in MoTe_2_ devices can reach up to 200 cm^−2^ V^−1^ s^−1^,^[^ ^9^, ^10^ ^]^ but in practice, the actual carrier mobilities of MoTe_2_ fall significantly below this theoretical range.^[^ ^4^, ^11^, ^12^ ^]^

Several techniques have been developed to improve the performance of 2D‐material‐based devices, including chemical doping, inducing strain, and substrate engineering.^[^ ^13^, ^14^, ^15^ ^]^ High dielectric constant (high‐κ) materials can also enhance the carrier mobility in 2D materials by strongly damping scattering from Coulombic impurities.^[^ ^16^, ^17^ ^]^ Atomic layer deposition (ALD) grown Al_2_O_3_ (κ = 9.1) is one of the widely used dielectric materials for 2D transistors due to its role to reduce defects and suppress charge trapping effects at the interface.^[^ ^18^, ^19^, ^20^ ^]^ Additionally, ALD‐grown Al_2_O_3_ serves as a passivation layer for 2D transistors to improve thermal and chemical stability. Moreover, the metal‐oxide dopes the 2D materials with n‐type carriers and elevates the electron current level. While possessing all these advantages, another potential aspect of this ALD layer is its ability to introduce tensile strain to the 2D material when the substrate is engineered in such a way that the weight of the passivated ALD layer and the thermal expansion difference between Al_2_O_3_ and MoTe_2_ exert tensile strain on the material, thereby enhancing device performance.

In this work, we first demonstrate a simple technique to improve the performance of MoTe_2_ FETs. Our approach involves creating a hole array in the substrate, using ALD‐grown Al_2_O_3_ as an additional dielectric layer on SiO_2_, and employing Al_2_O_3_ as a passivation layer for the MoTe_2_ FET. Our proposed structure induces tensile strain on MoTe_2_, leading to significant enhancement of the carrier mobility and on‐off ratio. The improved performance of the MoTe_2_ devices can be attributed to the significant damping of the Coulomb and electron–phonon scattering as a result of the change in the band structure of MoTe_2_. Additionally, the stable and good device quality leads to the metal–insulator transition (MIT) in the strained MoTe_2_ devices during temperature‐dependent electrical measurements, characterized by a sharp MIT critical point. This observation highlights the potential of MoTe_2_ for the development of fast‐switching devices and memory elements, with applications in efficient sensors, photodetectors, and energy storage devices.

## 2 Results and Discussion

**Figure 1:**
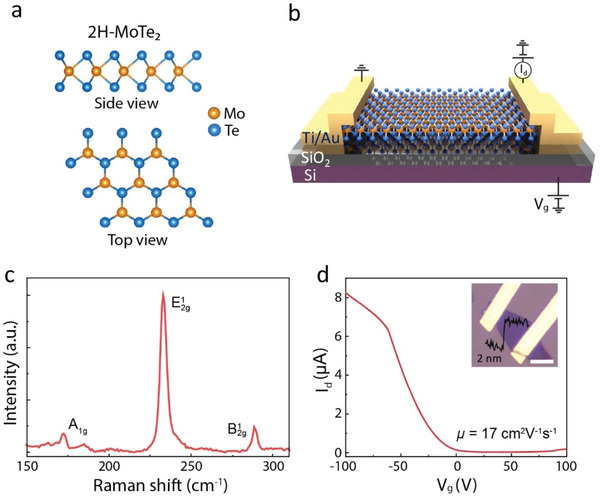
FET of 2H‐MoTe_2_ channel material. a) Illustration of the crystal structure of 2H‐MoTe_2_. b) Schematic of a back‐gated MoTe_2_ FET with Ti/Au electrodes on Si/SiO_2_ substrate. c) Room‐temperature Raman spectrum of a few‐layer MoTe_2_ and d) Transfer characteristics of the device with Ti/Au electrodes. The inset shows the device image with an atomic force microscopy (AFM) height profile of the flake with a scale bar of 5 µm.

**Figure** **1**a illustrates the crystal structure of 2H phase MoTe_2_, where a 2D hexagonal lattice structure is formed by sandwiching one layer of Mo atoms between two layers of S atoms. Relatively, 2H‐MoTe_2_ is thermodynamically more stable compared to other phases. Figure 1b presents a schematic of a FET on a typical Si/SiO_2_ substrate using a few‐layer MoTe_2_ as the channel material. The room‐temperature Raman spectrum of pristine MoTe_2_ obtained using a 532 nm (≈2.33 eV) laser excitation is depicted in Figure 1c. The distinctive Raman modes of MoTe_2_ appear at 173, 233, and 288 cm^−1^ corresponding to out‐of‐plane A_1g_, in‐plane $E_{2g}^{1}$, and bulk in‐active $B_{2g}^{1}$ modes, respectively.^[^ ^21^ ^]^ The transfer characteristics curve of a ≈2 nm thick MoTe_2_ FET at room temperature is shown in Figure 1d, revealing a p‐dominant ambipolar behavior. The calculated mobility (µ) of the device is 17 cm^2^ V^−1^ s^−1^. It is worth noting that, we observe a very thin layer (≈2 nm) of MoTe_2_ behaving as p‐type or p‐dominant ambipolar with Ti/Au electrodes. However, as the thickness of MoTe_2_ increases, the n‐branch of the transfer curve becomes stronger, leading to n‐type MoTe_2_ FET.

Usually, Al_2_O_3_ grown by ALD induces n‐doping in 2D materials when the metal oxide is used as a dielectric material and also as a passivation layer for 2D transistors. The effect of ALD doping is particularly noticeable in the electrical response of 2D FETs when compared with bare 2D FETs on Si/SiO_2_ substrate. Al_2_O_3_ dopes the material by injecting electrons in the channel, leading to a transition in carrier type from p‐type to p‐dominant, n‐dominant ambipolar, or even n‐type behavior. The µ_e_ in the devices also increases with an elevated electron current level. Meanwhile, Al_2_O_3_ contributes to a reduction in device hysteresis by minimizing carrier trapping and promoting a high‐quality interface. The high‐quality interface facilitated by Al_2_O_3_ may also enhance µ_h_ in the devices. Detailed discussion on the mechanism of doping by ALD‐grown Al_2_O_3_ is provided in the last section of this study.

**Figure 2:**
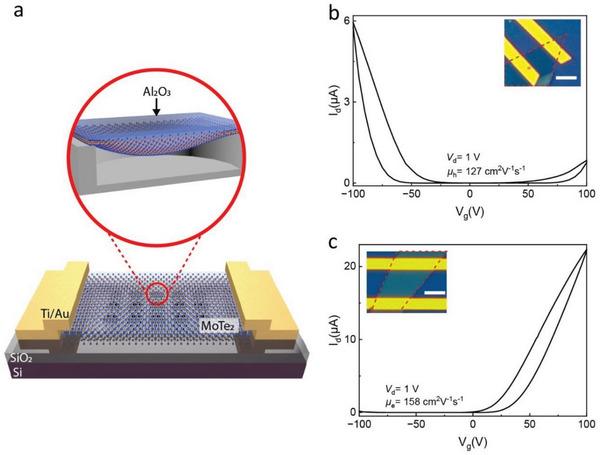
Electrical responses of strained MoTe_2_ FETs. a) A schematic of the device structure with a hole array in the substrate and Al_2_O_3_ passivation layer. b) The transfer curve of a strained  ≈2.7 nm thick MoTe_2_ exhibiting p‐dominant ambipolar device. The hole mobility (µ_h_) is presented in the figure. The inset shows an optical image of the device where the red dashed line defines the boundary of the flake. c) Transfer curve of the n‐type device with ≈5.8 nm thick MoTe_2_. The calculated electron mobility (µ_e_) is also shown in the image. The image of the device is shown in the inset of the figure. A drain voltage of *V*_d_ =  1 *V* is used for both of the transfer characteristic measurements. The scale bars in the inset of b) and c) are 3 µm.

In this experiment, we investigate the role of local strain on MoTe_2_ induced by hole‐array pattern on the Si/SiO_2_ substrate, while considering the ALD influences on the material. To achieve this, we fabricate hole arrays with various diameters on Si/SiO_2_, followed by deposition of 5 nm Al_2_O_3_ on the substrate. Subsequently, we transfer MoTe_2_ flakes onto the hole‐array substrate using the hot pick‐up technique.^[^ ^22^ ^]^ The adhesion between MoTe_2_ and ALD‐grown Al_2_O_3_ substrate relies on van der Waals bonding. The high‐quality and uniformity of layer‐by‐layer growth of Al_2_O_3_ in the ALD process enhances the adhesion. MoTe_2_ FETs with Ti/Au contacts are fabricated on the substrate, and an additional 50 nm layer of Al_2_O_3_ is deposited on the devices using a seeding layer to ensure uniform coverage on the MoTe_2_ because of the absence of dangling bonds on the surface of 2D materials (see the Experimental Section for more details). **Figure** **2**a shows a schematic of the device structure. The holes on the substrate are 200 nm in diameter and ≈50 nm in depth after the deposition of 5 nm Al_2_O_3_. Two different thicknesses of MoTe_2_ flakes are selected to analyze the strain effects: a ≈2.7 nm flake thickness exhibits p‐dominant ambipolar behavior, as shown in Figure 2b, while a thicker flake (≈5.8 nm) exhibits n‐type behavior. The transfer characteristics of the devices are presented in Figure 2c, with both devices exhibiting an on‐off ratio of 10^5^. The AFM measurement results of these samples are shown in Figure S2 (Supporting Information).

The 2‐probe field effect mobilities of these devices are calculated using the following equation

(1) $$\mu \;=\frac{L}{W}\;\;\left(\frac{1}{C_{{\mathrm{Al}}_{2}O_{3}}}+\frac{1}{C_{{\mathrm{SiO}}_{2}}}\right)\frac{1}{V_{d}}\;\frac{dI_{d}}{dV_{g}}\;$$

where L and W denote the length and width of the channel, respectively, $C_{{\mathrm{Al}}_{2}O_{3}}$ and $C_{{\mathrm{SiO}}_{2}}$ are the capacitances of Al_2_O_3_ and SiO_2_, respectively, *V*_d_ represents the drain voltage, and $\frac{dI_{d}}{dV_{g}}$ is the extrinsic transconductance of the devices. The thicknesses of the SiO_2_ and Al_2_O_3_ layers are 285 and 5 nm, respectively. The calculated µ_h_ of the device in Figure 2b is ≈127 cm^2^ V^−1^ s^−1^, while µ_e_ of the device in Figure 2c is ≈158 cm^2^ V^−1^ s^−1^. Note that the mentioned mobility values are underestimated due to the 2‐probe measurements, which include the effect of contact resistance. Accurate mobility numbers can be extracted from 4‐probe measurements.

**Figure 3:**
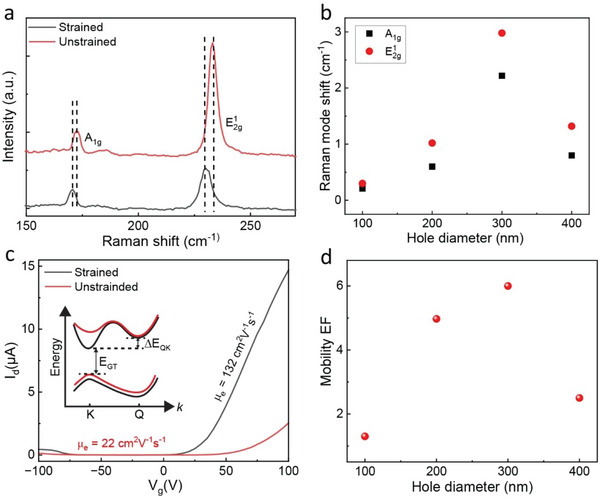
The hole‐array and top Al_2_O_3_ induced strain effects on MoTe_2_ FETs. a) Comparison of the Raman shifts of strained and unstrained samples. b) Variation in the Raman peak shifts with different hole diameters. c) Change in the current and µ_e_ of the devices with and without strain. Inset illustrates the simplified band diagram where *E*_GT_ and Δ*E*_QK_ refer to the bandgap and the gap between the Q and K point of the conduction band after application of tensile strain, respectively. Note that the energy bands with red colors correspond to unstrained MoTe_2_ and the black lines correspond to bands of strained MoTe_2_. d) Mobility enhancement factor (EF) of the devices as a function of hole diameters.

In order to get a deeper understanding, we create hole arrays in the substrate with different diameters, such as 100, 200, 300, and 400 nm. The target depth of the holes is 70 nm; however, the actual depth ranges from 40 to 55 nm after 5 nm Al_2_O_3_ deposition (for details see the Experimental Section). The as‐transferred flakes of MoTe_2_ having a thickness of ≈5 nm, are suspended on the hole arrays experience relatively small strain. To enhance the localized tensile strain on the MoTe_2_ flakes, we grow a 50 nm layer of Al_2_O_3_ using the ALD process. As we know that Raman spectroscopy is a well‐established tool to investigate the stain in 2D materials, we use a 532 nm laser to characterize the nature and magnitude of strain in both strained and unstrained MoTe_2_. The distinct Raman peaks observed in both samples confirm the intact and high‐quality of the flakes. We notice that the Raman modes of strained MoTe_2_ are redshifted compared to the unstrained regions of the flake, as shown in **Figure** **3**a. Additionally, the intensities of the Raman peaks are decreased in the strained samples. These findings are consistent with previous reports.^[^ ^23^, ^24^, ^25^ ^]^ Especially, in our samples, most strained samples exhibit a redshift of ≈2.98 and ≈2.22 cm^−1^ for prominent $E_{2g}^{1}$ and A_1g_ modes, respectively. Notably, in the previous reports,^[^ ^23^, ^24^, ^25^ ^]^ the shift in the A_1g_ mode under tensile strain was almost negligible. We notice a significant shift in the A_1g_ mode of strained MoTe_2_. We assume that the shift is the result of using a few‐layer and unsupported MoTe_2_ or the effect of change in the dielectric environment^[^ ^26^ ^]^ in the hole‐array regions of our samples. Another major reason behind this larger shift in A_1g_ could be the result of the high level of doping in MoTe_2_ induced by Al_2_O_3_.^[^ ^25^, ^27^, ^28^ ^]^ Figure 3b illustrates the effect of hole diameter variation on the shift of each Raman mode in strained MoTe_2_ samples compared to the unstrained samples. It is evident that the shift of all Raman peaks is increasing with the increase in the hole diameter, with the highest shift observed when the diameter is 300 nm. This indicates that the 300 nm hole diameter exerts the strongest tensile strain on the MoTe_2_ flake around the peripheral region of the hole. The amount of strain approximated from the shift of $E_{2g}^{1}$ mode is ≈0.67%.^[^ ^23^ ^]^ The strain in the material decreases at 400 nm, which could be attributed to the increased flat or less strained region at the center of the hole.

Next, we analyze the influence of the hole‐array pattern and top Al_2_O_3_‐induced strain in the MoTe_2_ device performance. Local tensile strain resulting from our proposed structure enhances the electron current and mobility of MoTe_2_ FETs by up to ≈6 times compared to the unstrained flat sample. Figure 3c shows the comparison of the current and µ_e_ of the strained and unstrained samples. The optical image and height profile of the MoTe_2_ flake are presented in Figure S3 (Supporting Information). The performance enhancement in our devices could be attributed to the reduced electron–phonon scattering due to the band structure changes upon application of the tensile strain to MoTe_2_.^[^ ^15^, ^29^ ^]^ A schematic of a simplified band structure of MoTe_2_ before and after strain is presented in the inset of Figure 3c. The red and black colored energy bands in the figure correspond to unstrained and strained few‐layer MoTe_2_. The bandgap *E*_GT_ of MoTe_2_ decreases as the valley of the conduction band at the K point is lowered when tensile strain is applied to the few‐layer MoTe_2_ flake.^[^ ^29^ ^]^ As a result, the energy difference between K point and Q point (denoted as Δ*E*_QK_) increases, resulting in very low electron intervalley scattering by reducing the electron effective mass.^[^ ^15^ ^]^ This could be the major reason for the enhancement of µ_e_ in MoTe_2_ devices. In addition, the contact resistance of the devices decreases by reducing the Schottky barrier height in the devices when tensile strain is applied to 2D materials, also leading to the high µ_e_.^[^ ^30^, ^31^ ^]^ Among the 2D TMDCs, MoTe_2_ is more susceptible to defect formation and charge trapping.^[^ ^7^ ^]^ The application of tensile strain to MoTe_2_ could potentially enhance the carrier lifetime by mitigating the trapping of the charge carriers.^[^ ^32^, ^33^ ^]^ This could also lead to high current and mobility in the devices.

Figure 3d illustrates the hole diameter‐dependent mobility EF. The mobility increases with increasing hole diameter up to 300 nm, which also corresponds to the highest mobility EF. A close value of the EF is also noticed at 200 nm diameter. The EF decreases when the hole diameter increases to 400 nm. This trend in the mobility EF is analogous to the hole diameter‐dependent strain observed in the samples presented in Figure 3b. From these results, it is apparent that the tensile strain induced by the hole‐array pattern in the substrate and top Al_2_O_3_ layer enhances the MoTe_2_ device performance significantly.

High‐κ dielectric materials improve the device performance by strongly reducing Coulomb scattering^[^ ^16^, ^17^ ^]^ and enhancing gate capacitance.^[^ ^19^ ^]^ An additional layer of Al_2_O_3_ on SiO_2_ has a similar influence on the device performance. Al_2_O_3_ provides strong gate controllability and reduces the Schottky barrier height (SBH) between metal and semiconductor, resulting in increased carrier mobility through the thermionic emission process.^[^ ^20^ ^]^

**Figure 4:**
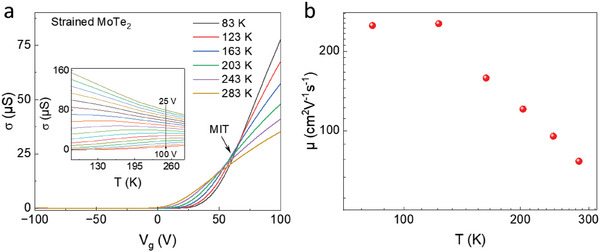
Temperature‐dependent transport and carrier mobility of strained MoTe_2_ FET. a) Variation in conductance (σ) for different values of *V*_g_. Inset shows the temperature‐dependent σ of the device. b) Change of carrier mobility µ as a function of temperature.

For further analysis of the Schottky barrier, we investigate the transfer characteristics of the devices at different temperatures. **Figure** **4**a shows the transfer curves of a n‐type MoTe_2_ transistor made with Ti/Au contacts at various temperatures. Remarkably, we observe MIT in MoTe_2_ devices. The drain current, measured as a function of gate voltage, shows a crossover at a specific gate voltage. This crossover point, also known as critical or MIT point is at ≈*V*_g_ = 62 V. Below the point, the MoTe_2_ device behaves as an insulator, as device conductivity increases with temperature increase. Meanwhile, above the MIT point, the conductivity decreases with increasing temperature, behaving as a metal. At the MIT point, the carrier density is estimated to be *n*_D _ ≈  3.74 × 10^−12^ cm^−2^, calculated using $n_{D}=\frac{C_{\mathrm{ox}}\left(V_{g}-V_{\mathrm{th}}\right)}{q}\;$, where *C*_ox_ is the overall capacitance of the dielectrics, *V*_th_ is the threshold voltage, and *q* is the charge of an electron. The dependency of the conductivity on the temperature is shown in the inset of Figure 4a. In 2D materials, temperature‐dependent transport in the channel is typically divided into two different regimes, separated by the mobility or conduction band edge *E*_c_.^[^ ^34^, ^35^, ^36^ ^]^ In the insulating regime, the Fermi energy *E*_F_ moves away from the mobility edge which results in strong localization of the charge carriers. Therefore, the conductivity increases with the increase in the temperature due to thermal activation. Conversely, *E*_F_ moves toward *E*_c_ in the metallic regimes, and charge carriers are weakly localized. As the temperature increases, phonon scattering becomes more dominant, leading to a decrease in the conductivity in this regime.^[^ ^37^, ^38^ ^]^

The variation of mobility of the device with temperature is shown in Figure 4b. The mobility is ≈250 cm^2^ V^−1^ s^−1^ at 80 K, indicating strong screening of Coulomb scattering due to the low temperature and thin Al_2_O_3_ dielectric layer. The mobility of the transistor decreases with the temperature increase as the phonon scattering becomes dominant over Coulomb scattering. The hysteresis in the device increases with the temperature increase as well; however, the value of hysteresis is very small, even at room temperature compared to the FET made solely on flat Si/SiO_2_ substrate, as shown in Figure S5 (Supporting Information). This indicates the higher quality of the layers of Al_2_O_3_ than SiO_2_ in terms of low surface defects resulting from the layer‐by‐layer deposition of Al_2_O_3_ in the ALD process. This results in less carrier trapping during the conduction.

**Figure 5:**
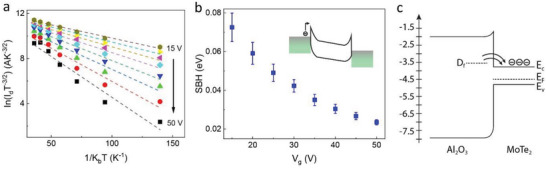
Effect of ALD Al_2_O_3_ on strained MoTe_2_ FET performance. a) Arrhenius plot as per thermionic emission model at different gate voltages. b) The extracted Schottky barrier heights (SBHs) at various *V*_g_. c) ALD Al_2_O_3_ induced doping mechanism.

From the temperature‐dependent transport measurements of MoTe_2_ devices, we can also analyze the quality of the contacts (Ti/Au) on the MoTe_2_ channel while considering the influence of top‐bottom Al_2_O_3_. The Arrhenius plot in **Figure** **5**a is obtained from the thermionic emission model, $I_{d}\propto T^{\frac{3}{2}}.\exp\left[-q\phi_{B}/k_{B}T\right]$, where ϕ_B_ represents the Schottky barrier height (SBH) and *k*_B_ is the Boltzmann constant. The extracted SBH as a function of *V*_g_ is presented in Figure 5b with a schematic of the thermionic emission of the electrons over the SBH in the inset. The low value and nearly linear dependence on *V*_g_ of the SBH indicate thermionic emission of the carriers contributing to the current and leading to high mobility in the devices. This could be the result of the combined effect of the top Al_2_O_3_ used for inducing strain and the bottom Al_2_O_3_ used as an additional dielectric layer for MoTe_2_ FETs. Understanding the mechanism of doping in MoTe_2_ by ALD‐grown Al_2_O_3_ can provide further insight into the enhancement of device performance.

The n‐type doping in 2D materials by ALD‐grown Al_2_O_3_ is mainly attributed to the presence of oxygen vacancies in Al_2_O_3_.^[^ ^39^, ^40^ ^]^ These oxygen vacancies create defect states within the bandgap of Al_2_O_3_ as shown in Figure 5c.^[^ ^41^ ^]^ Depending on the electron occupation, each of the defect states has a charge and an energy level. At the interface of oxide‐MoTe_2_, if the defects occupy energy levels above the MoTe_2_ conduction band and donate their electrons to become positively charged, then MoTe_2_ becomes n‐doped, leading to high density and mobility of the electrons in the channel. The presence of Al_2_O_3_ both below and above MoTe_2_ enhances the doping effect, thus resulting in improved device performance.

## 3 Conclusion

In summary, we demonstrate a simple and effective method to modulate the carrier type and enhance the performance of few‐layer MoTe_2_ FETs. The combination of the hole‐array pattern in the substrate and ALD‐grown Al_2_O_3_ layers as an additional dielectric and passivation layer enables us to achieve significant enhancements in both the hole and electron mobility of MoTe_2_ devices. The current and the electron mobility of our MoTe_2_ devices are increased by up to 6 times due to the application of tensile strain on MoTe_2_ by the proposed structure. The strain applied to MoTe_2_ changes the band structure of the material and reduces the electron‐phonon scattering in the samples, leading to high electron mobility in the devices. Additionally, we observe MIT in MoTe_2_ during analyzing the temperature‐dependent behavior of the devices. Overall, our proposed structure represents a promising approach for advancing the performance of MoTe_2_ devices for future electronics.

## 4 Experimental Section

### Hole‐Array Substrate

For making hole arrays, a p‐doped silicon substrate (0.001–0.005 Ω cm) covered with a 285 nm thick thermally grown SiO_2_ was cleaned with acetone and IPA. Patterns with different diameters of circles were created by electron beam lithography (EBL Vistec, EPBG 5000) after coating the substrate with PMMA A4. Then the substrate was subjected to a reactive ion etching (RIE) tool (Plasmalab 80 Plus, Oxford Instruments) for SiO_2_ etching. CHF_3_ and O_2_ were used as process gases and the rate was optimized to get 40 ± 5 nm min^−1^ etch rate. The etching was carried out for 2 min to get 70 nm of SiO_2_ etching from the PMMA opening. After etching the PMMA was dissolved in acetone.

### Al_2_O_3_ Growth in ALD

The substrates with hole arrays were inserted in the ALD tool (Beneq TFS‐500). A 5 nm Al_2_O_3_ was grown on the hole‐array substrate using trimethylaluminium (TMA) and water at 200 °C, while the process parameters were optimized to deposit Al_2_O_3_ with 0.1 nm per cycle rate. Another deposition of Al_2_O_3_ was carried out after device fabrication. However, this time before growing the oxide, a 2 nm Al was deposited as a seed layer using e‐beam evaporation on the samples because MoTe_2_ has no dangling bond on the surface, therefore uniform growth of Al_2_O_3_ on 2D materials requires this seeding layer. After that, a 50 nm Al_2_O_3_ was grown using the same growth method mentioned before.

### Device Fabrication

The few‐layer MoTe_2_ flakes were mechanically exfoliated from the commercially available MoTe_2_ crystal (2D Semiconductors) on a p‐doped silicon substrate (0.001–0.005 Ω cm) covered with a 285 nm thick thermally grown SiO_2_. A custom‐built 2D transfer setup was used to prepare the samples. After exfoliation, electron beam lithography (EBL Vistec, EPBG 5000) and metallization were carried out by using electron beam evaporator (MASA, IM‐9912) under ≈10^−7^ torr chamber pressure to deposit a 5 nm Ti adhesion layer followed by 50 nm thick Au. The height of the flakes were determined using an atomic force microscope (Bruker, Dimension Icon).

### Raman Spectroscopy

The room‐temperature Raman spectra were collected in back‐scattering geometry with a confocal micro‐Raman system (WITec alpha300 RA+). Samples were excited using a 532 nm laser with a spot size of less than 1 µm (×100 objective, 0.9 NA). Low laser power (<500 µW) was used to avoid laser‐induced damage in the samples.

### Electrical and Temperature‐Dependent Measurements

All the electrical measurements were carried out with a custom‐built setup based on a Linkam LN600‐P probe station with environmental and temperature controller (T96‐S) using a source‐measure unit (Keithley 2400) and a Keithley 2700 multiplexing/voltage measurement unit. The temperature measurement system could achieve a 150 °C min^−1^ heating rate and 100 °C min^−1^ cooling rate with < 0.1 °C temperature stability. All the measurements were repeated twice at each point to get confident data.

## Conflict of Interest

The authors declare no conflict of interest.

## Supporting information

Supporting Information

## Data Availability

The data that support the findings of this study are available from the corresponding author upon reasonable request.
