# Supplementary material for: Strain Engineering for Enhancing Carrier Mobility in MoTe2 Field‐Effect Transistors
Source: Adv Sci (Weinh). 2023 Aug 8;10(29):2303437. doi: 10.1002/advs.202303437 (PMC10582429; doi:10.1002/advs.202303437)
Supplement: Supplementary file 1 — Supporting Information [file ADVS-10-2303437-s001.pdf]

ADVANCED SCIENCE
Open Access

Supporting Information

for *Adv. Sci.*, DOI 10.1002/advs.202303437

Strain Engineering for Enhancing Carrier Mobility in $MoTe_2$ Field-Effect Transistors

Abde Mayeen Shafi, Md Gius Uddin, Xiaoqi Cui, Fida Ali, Faisal Ahmed, Mohamed Radwan, Susobhan Das, Naveed Mehmood, Zhipei Sun and Harri Lipsanen**

Supporting Information

Strain Engineering for Enhancing Carrier Mobility in $MoTe_2$ Field-Effect Transistors

Abde Mayeen Shafi,[*] Md Gius Uddin, Xiaoqi Cui, Fida Ali, Faisal Ahmed, Mohamed Radwan, Susobhan Das, Naveed Mehmood, Zhipei Sun, Harri Lipsanen[*]

A. M. Shafi, M. G. Uddin, X. Cui, F. Ali, F. Ahmed, M. Radwan, S. Das, N. Mehmood, Z. Sun, H. Lipsanen
Department of Electronics and Nanoengineering
Aalto University
Tietotie 3, FI-02150, Finland
E-mail: abde.shafi@aalto.fi, harri.lipsanen@aalto.fi

Prof. Z. Sun
QTF Centre of Excellence, Department of Applied Physics
Aalto University
Aalto FI-00076, Finland

Table of contents:

1: Atomic force microscopy (AFM) of $MoTe_2$ devices on hole-array. 3

2: Effect of Al_2O_3 passivation on $MoTe_2$ field effect transistor (FET).. 6

3: Hysteresis in temperature-dependent transfer characteristics of a strained $MoTe_2$ FET...6

1: Atomic force microscopy (AFM) of $MoTe_2$ devices on hole-array.

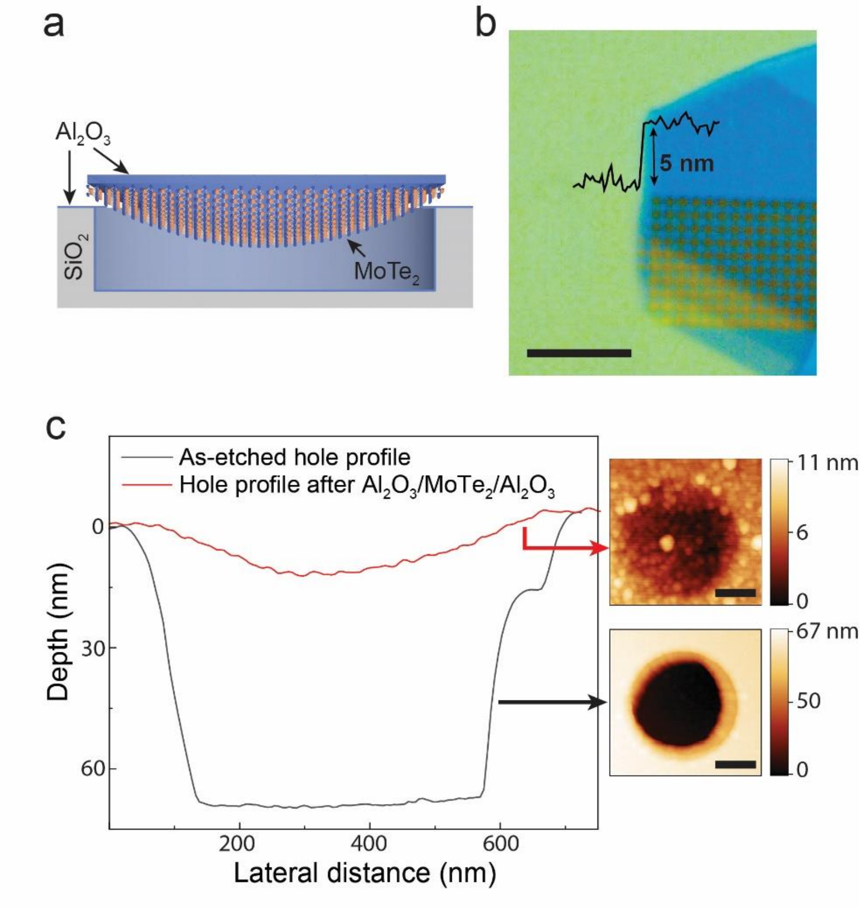

Figure S1: Morphological characterization of our devices. **(a)** Schematic representing the cross-section of the devices. **(b)** Optical image of a ~5 nm thick $MoTe_2$ flake transferred onto hole-arrays. Scale bar: 5 µm. **(c)** Simplified depth profiles of as-etched hole array and hole profile after transfer for the $MoTe_2$ flake and the 50 nm Al_2O_3 deposition. The corresponding AFM images of the depth profiles are indicated with red and black arrows. Scale bars for both images are 100 nm.

The hole arrays for our strain engineered $MoTe_2$ devices are fabricated using electron beam lithography (EBL). We use Si wafers with a 285 nm thick thermally grown SiO_2 as capping layer. First, we spin coat the Si/SiO_2 chips with PMMA A4 positive EBL resist at 2000 rpm for 1 min. Subsequently, the sample is baked on a hot plate at 160 ˚C for 2 min. Resist thickness on chip is calculated as ~280 nm.

To create arrays of holes of 300 nm diameter and 400 nm pitch, we use a dose of 1200 $\mu C/cm^2$ and a current of 1 nA during EBL patterning. Then the electron beam-exposed sample is developed in an MIBK: IPA (1:3) solution for 30 s, resulting in an anisotropic hole profile into the resist. A diameter deviation of ±20 nm is considered due to back-scattered electrons from the EBL exposure. We achieve a controlled SiO_2 etch rate of 40±5 nm/minute using an optimized RIE recipe (CHF_3:O_2=45:5, 200 W, 1 min) at room temperature. After

fabricating hole arrays with desired parameters, we grow a 5 nm thick Al_2O_3 by atomic layer deposition (ALD) on the as-etched Si/SiO_2 substrate. Few-layer $MoTe_2$ flakes are transferred onto the fabricated hole-arrays using PPC/PDMS -assisted hot pick up method.[1] This transfer method is useful to prevent excessive pressure exerted on the $MoTe_2$ flake during transfer compared with only PDMS or scotch-tape based transfer methods that could potentially tear the flake. Following the flake transfer, we grow a 50 nm thick layer of Al_2O_3 employing the ALD technique.

As presented in Fig. S1, high-resolution AFM measurements reveal the topography of our devices. Figure S1(a) and (b) show the cross-section schematic and optical image of a few-layer $MoTe_2$ flake transferred onto a hole-array, respectively. In Figure 2(c), the depth profiles of an as-etched hole (black line) and hole/$MoTe_2$/Al_2O_3 (50 nm) structure (red line) are presented, with the depth of $MoTe_2$ suspended on hole structure measuring ~11 nm. Corresponding AFM images of the as-etched hole and the hole/$MoTe_2$/Al_2O_3 (50 nm) structure are provided on the right panel of Figure S1(c), indicated with black and red arrows, respectively.

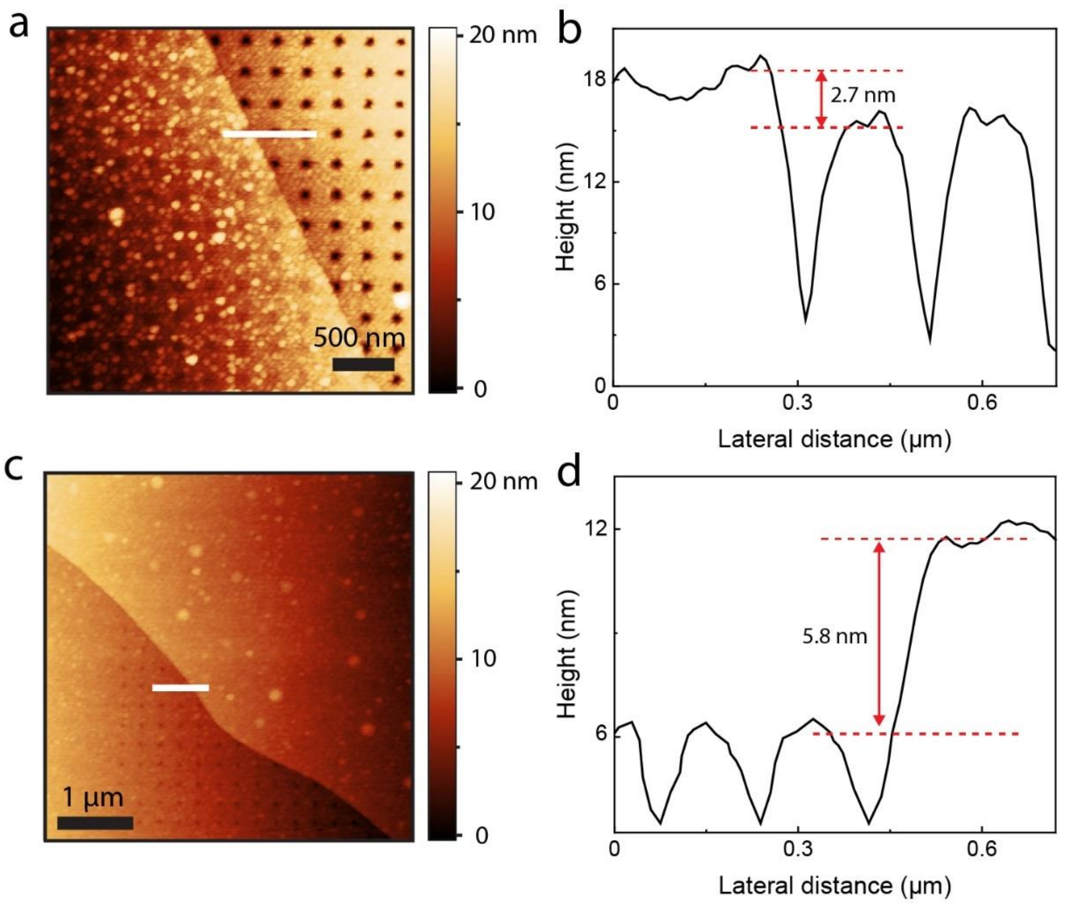

Figure S2: AFM measurements of $MoTe_2$ devices. **(a)** and **(b)** show the AFM image and thickness profiles of 2.7 nm thick strained $MoTe_2$ flake, respectively. **(c)** and **(d)** are the AFM image and thickness profiles of 5.8 nm thick strained flake, respectively. The thickness profiles of the flakes are measured from the white lines shown in the images.

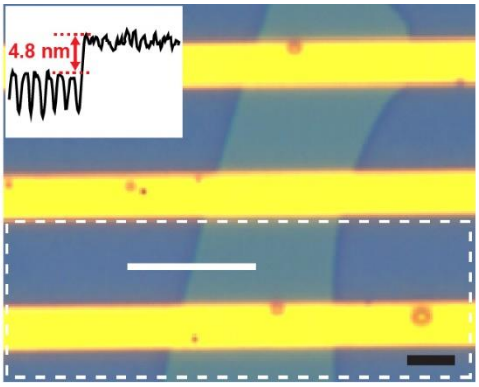

Figure S3: Optical mage of the flake used for comparing $MoTe_2$ mobility enhancement factor with and without strain. The while dashed box corresponds to the part of the flake with hole-array patterned substrate. The diameter of the holes for creating strain in this sample is 200 nm. Inset shows the thickness profile of the flake taken from the white line in the optical image. Scale bar: 2 μm.

2: Effect of Al_2O_3 passivation on $MoTe_2$ field effect transistor (FET).

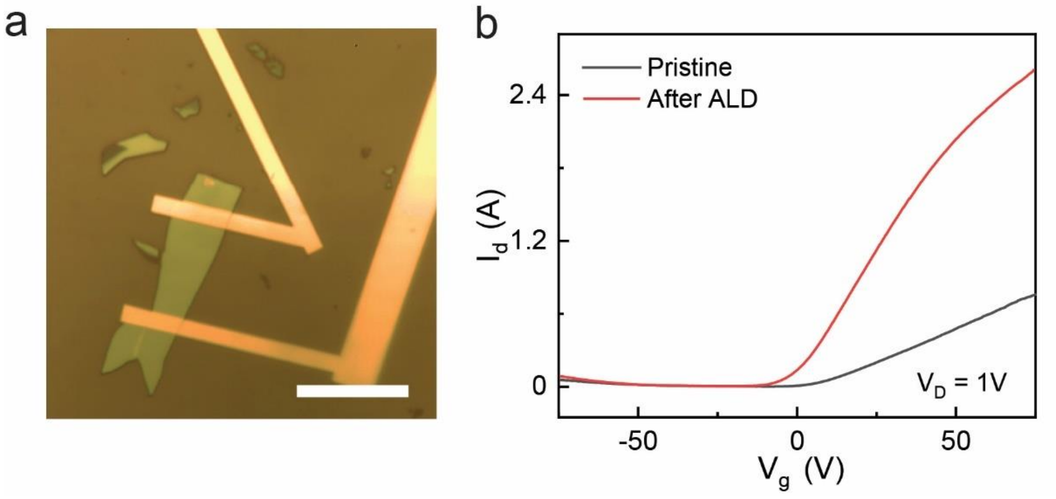

Figure S4: The doping effect of ALD-grown Al_2O_3 on a $MoTe_2$ device. **(a)** Optical image of the device after Al_2O_3 passivation. Scale bar: 10 µm. **(b)** Transfer curve of the device before and after ALD. The introduction of ALD-grown Al_2O_3 passivation on the device demonstrates an increase in carrier transport compared to the device without Al_2O_3.

3: Hysteresis in temperature-dependent transfer characteristics of a strained $MoTe_2$ FET.

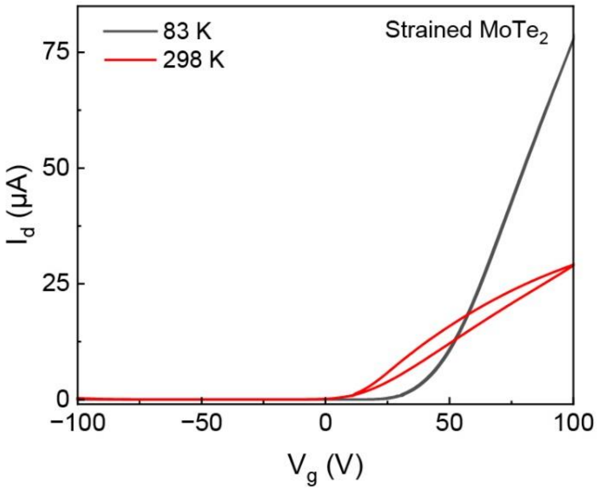

Figure S5: Hysteresis of a strained $MoTe_2$ device at 83 K and 298 K, where the calculated hysteresis at 83 K is negligible and ~12 V at room temperature.

References

[1] F. Pizzocchero, L. Gammelgaard, B. S. Jessen, J. M. Caridad, L. Wang, J. Hone, P. Bøggild, T. J. Booth, *Nat. Commun.* **2016**, *7*, 11894.
